# Retinoblastoma patients treated in Sri Lanka from 2014 to 2020: epidemiology, clinical status and correlates of lag time in seeking tertiary care services

**DOI:** 10.1186/s12886-024-03541-3

**Published:** 2024-07-17

**Authors:** Nirosha Kugalingam, Deepthi de Silva, Hiranya Abeysekera, Sriyani Nanayakkara, Shamala Tirimanne, Vishvanath Chandrasekharan, Pushpa Lalani Jayawardana

**Affiliations:** 1 Department of Chemistry, Faculty of Science, University of Colombo, Colombo, Sri Lanka; 2 Department of Physiology, Faculty of Medicine, University of Kelaniya, Kelaniya, Sri Lanka; 3 Lady Ridgeway Hospital, Colombo, Sri Lanka; 4 National Eye Hospital, Colombo, Sri Lanka; 5 Department of Plant Sciences, Faculty of Science, University of Colombo, Colombo, Sri Lanka; 6 Sri Lanka Institute of Biotechnology, Pitipana, Homagama, Sri Lanka; 7 Department of Public Health, University of Kelaniya, Kelaniya, Sri Lanka

**Keywords:** Retinoblastoma, Staging, Mortality, Early screening for RB, Lag time for tertiary care

## Abstract

**Background:**

Retinoblastoma (RB) is a tumour of children < 5 years with a incidence of 1 in 20,000. Around 20 RB cases are diagnosed yearly in Sri Lanka, a lower middle-income country with high literacy levels and healthcare free at point of delivery. Incidence, local and systemic severity and mortality related to RB are reportedly high in low- and middle- income countries in comparison to higher income countries. Aims of this study were to describe demographic, socioeconomic, and clinical characteristics of Sri Lankan RB patients attending the designated RB unit at the Lady Ridgeway Hospital (LRH), Colombo between January 2014 to December 2020, and determine correlates of lag time (LT) for first tertiary care visit after detecting the first symptom/sign.

**Methods:**

Two descriptive cross-sectional studies (DCSS) were conducted, one on 171 RB patients with demographic and clinical data collected between 2017 and 2020. In 2021, the second DCSS took place where socioeconomic and further demographic data were collected using telephone interviews, recruiting a subgroup of 90 (53%), consenting and contactable RB patient/ parent pairs. Bivariate and multivariable analyses were applied to determine correlates of LT of > 4 weeks for first tertiary care visit. Results were expressed as odds ratios and 95% confidence intervals.

**Results:**

**LRH survey (*N* = 171)**: Median age at diagnosis was 15 months (range 1–94 months; IQR: 8–27); 89 (52%) were females. Groups D and E tumours were 25.7% (*n* = 44) and 62.6% (*n* = 107) respectively with 121 (71%) enucleations. The number of deaths were 2 (1.2%). **Telephone survey (*N* = 90)**: Proportion with LT of > 4 weeks for first tertiary care visit was 58% (*n* = 52). None of the putative risk factors (ethnicity, parental educational level, socioeconomic status, distance from residence to tertiary care unit and receiving financial assistance) were associated with LT in both analyses.

**Conclusion:**

Despite a high proportion with groups D and E tumours and enucleations, mortality rate was low, most likely due to availability of designated tertiary care. No correlates for LT of > 4 weeks for tertiary care presentation were identified. Early RB detection needs rigorous implementation of screening strategies and increased awareness among primary care health workers and parents.

**Supplementary Information:**

The online version contains supplementary material available at 10.1186/s12886-024-03541-3.

## Introduction

Retinoblastoma (RB) is a rare, primary malignant tumour which occurs in the embryonic retina due to mutations in the *RB1* gene [1, 2]. It is the most common solid tumour of infancy and childhood with a global incidence of one case per 15,000–20,000 live births [3]. Inactivation of both copies of *RB1* in the cells of the retina is associated with the development of RB. In 60 − 65% of RB patients, the disease is ‘sporadic’ with inactivating *RB1* variants located only in the retina. There is a lower risk of RB recurrence in the patient and their siblings [3]. Germline (inherited) variants of *RB1* are identified in around 85% of patients with bilateral RB and around 15% of unilateral RB patients.

The main presentations of RB are leukocoria (white pupillary reflex), strabismus [squint] or both [3–6]. Leukocoria is observed by a trained observer by shining an ophthalmoscope light on the eye to detect the pathological white glow, instead of the normal red reflex in a healthy eye [7]. It is occasionally observed in photographs of affected children, usually at an advanced stage of disease [7, 8]. The International Intraocular Retinoblastoma Classification [IIRC] is the most widely used classification system for RB, from the earliest stage A to late stage E. It is based on tumor size, location, presence of associated seeding and/ or retinal detachment [9]. Late detection of RB leads to visual and systemic morbidity and mortality [4].

Sri Lanka is an Indian Ocean island with a population of 21.8 million and a GDP per capita of US dollars 3852 (in 2019), making it a lower-middle income country, although the recent, severe economic crisis will adversely impact its GDP. Sri Lanka has a high literacy rate (97%) and a health service which is free at the point of delivery. There is a well organised, universal maternal and child public health care system, delivered at home by the Public Health Midwife (PHM), under the supervision of a Medical Officer of Health (MOH), providing grassroot level services from birth to five years. Access to hospital-based medical care is available for more severe illness and is free at the point of delivery. Parents can also seek privately funded health care and there is a parallel system of traditional medicine [10]. There are around 61 consultant level ophthalmologists in the government sector and around 20 ophthalmologists in the private sector hospitals in Sri Lanka. The treatment of RB was originally undertaken at different centres in the country, but since 2014, all suspected and affected RB patients have been managed at a single, tertiary care ophthalmology unit at the Lady Ridgeway Hospital (LRH), a paediatric hospital in the capital city of Colombo. Referrals to the RB unit has many sources including other ophthalmologists, paediatricians, MOH or by parents directly attending the outpatients’ unit at the LRH, from where they can be referred to the ophthalmology unit.

To the best of our knowledge, this is the first epidemiological study on RB in Sri Lanka. The objectives of this study were to describe demographic, socio-economic and clinical characteristics of RB patients attending the LRH between January 2014 to December 2020, and to determine correlates of lag time for first attendance at the tertiary care unit.

## Materials and methods

This paper is based on two descriptive cross-sectional studies (DCSS). The data for the first DCSS were based on data collected retrospectively using archived medical records maintained at the LRH for RB patients admitted between January 2014 to December 2017. Data collection from patients admitted between 2018 and 2020 took place prospectively (following commencement of this research project). Data obtained by review of medical records included name, gender, date of birth, home address, family history, age at diagnosis, laterality of the tumour/s, IIRC based tumour stage and treatment modalities (Supplementary material 1).

The second DCSS was conducted in 2021. It was meant to include the entire cohort of 171 RB patient/parent pairs. However, only 90 were contactable and all identified patient/parent pairs consented to participate in this survey, giving a response rate of 52.6%. An open and close ended questionnaire, comprising 30 items (Supplementary material 2) was administered, and the interview lasted between 20 and 30 min. The information obtained included more detailed demographic, socioeconomic and other relevant information including number of people in the household, parental education and employment, distance to the LRH, type of transport, time needed to travel to hospital and relevant clinical details, including the duration between detection of first symptom by the parent/s, and first visit to the tertiary care unit. Distance from home to LRH was calculated using Google maps according to the confirmed address given by the parents. All the interviews were conducted in the parents’ language of preference.

Data were recorded in a data sheet which was anonymized and transferred to a computerized database. Both the paper and computer records were accessible only to members of the research team to ensure confidentiality. The missing data was obtained when patients and parents attended for follow up at the LRH.

### Data analysis

The quantitative data (ages of RB patients and parents, duration between observing first sign and seeking tertiary care) were tested with the Shapiro-Wilk test to determine normality. As these variables did not conform to a normal distribution, summary statistics were based on median (interquartile range [IQR]) and the range. Qualitative data were computed as proportions and expressed as percentages.

A cut off value of > 4 weeks of lag time was arbitrarily considered for the analytical component of the study. The chi squared test was applied for bivariate analysis and multivariable analysis to control for confounding factors, using multiple logistic regression. The software used for the statistical analysis was Statistical Package for the Social Sciences (SPSS) version 20 and Winpepi version 11.65.

## Results

**Demographic and socioeconomic features of the cohort of 171 RB patients retrieved from the medical records at the LRH (*N* = 171) and telephone survey (*N* = 90)** [Table 1].

### Demographic features obtained from the LRH survey (Table 1A)

#### Age (*N* = 171)

The median age of the 171 patients at first visit to LRH was 15 months (IQR: 8–27) with a range of 1–94 months. Unilateral RB patients had a median age of 18 months (IQR: 11–36) with a range of 1–94 months whereas that of bilateral patients was 8 months (IQR: 5–15) with a range of 2–36 months. Highest proportion (35.7%; *n* = 61) among the total RB patients was in the age group of 00–10 months. The highest proportions of unilateral (31.7%; *n* = 38) and bilateral (62.7%; *n* = 32) patients were between 11–20 and 00–10 months respectively.

#### Gender (*N* = 171)

The proportion of females among the cohort was 52.0% (*n* = 89).

#### Residential area by province (*N* = 171)

The patients were distributed among all the nine provinces of the country. The highest proportion (34.5%; *n* = 59) was from the Western province and the lowest (4.7%; *n* = 8) from the Northern province (Table 1). The rates per population of RB cases ranged from 5 per 1,000,000 in the Central province to 10 and 11 by 1,000,000 in the Western and Uva provinces respectively.

### The demographic and socioeconomic results based on the telephone survey (Table 1B)

#### Ethnicity (*N* = 90)

A majority of the patients (76.7%; *n* = 69) were Sinhala which is almost similar to the ethnic distribution for Sri Lanka [11].

#### Parental age (years) at the birth of the index child (*N* = 90)

The median age of the fathers was 33 years (IQR: 31–36) with a range of 20–46 years whereas that of mothers was 29 years (IQR: 25–32), with a range of 17–39 years. 72% (72.2%, *n* = 65) of fathers and 61.1% (*n* = 55) mothers were in the age groups of 31–40 and 21–30 years respectively.

#### Parental educational status (*N* = 90)

The proportion of fathers and mothers whose educational status was below General Certificate of Education Ordinary Level was 15.6% ((*n* = 14) and 11.1% (*n* = 10) respectively. The proportion of fathers and mothers who have attained tertiary education was 5.6% (*n* = 5) and 14.4% (*n* = 13) respectively.

#### Parental occupational status (*N* = 90)

Among fathers, a majority (66.7%; *n* = 60) were employed in occupations related to skill level 2 where as a majority (80.0%; *n* = 72) of mothers were homemakers [12].

#### Total family income (*N* = 90)

A proportion of 27.8% (*n* = 25) had an income of > Rupees 50,000, whereas the second highest proportion of 26.7% (*n* = 24) were earning between Rupees 10,001–20,000.

#### Receiving financial assistance (*N* = 90)

A proportion of 21.1% (*n* = 19) were recipients of financial assistance.

#### Distance from residence to LRH (*N* = 90)

The highest proportion (45.6%; *n* = 41) of the RB patients were residing more than 50 km away and the second highest proportion were residing between 5.1 and 10 km.

**Table 1:** Demographic and socio-economic characteristics of retinoblastoma (RB) patients related to first visit to RB tertiary care unit, Lady Ridgeway Hospital (LRH), based on the medical records (MR) at LRH and telephone survey (TS)

1 A.	Demographic & socioeconomic features obtained from clinical records at LRH during first visit	
	Variable	Frequency
**01.**	**Age (months) of RB patients**	
**a.**	**Total sample (** ***N*** ** = 171)**	
	00–10	61 (35.7%)
	11–20	51 (29.8%)
	21–30	23 (13.4%)
	31–40	14 (08.2%)
	> 41	22 (12.9%)
**b.**	**Unilateral cases (** ***n*** ** = 120; 70.2%)**	
	00–10	29 (24.2%)
	11–20	38 (31.7%)
	21–30	18 (15.0%)
	31–40	13 (10.8%)
	> 41	22 (18.3%)
**c.**	**Bilateral cases (** ***n*** ** = 51; 29.8%)**	
	00–10	32 (62.7%)
	11–20	13 (25.5%)
	21–30	05 (09.8%)
	31–40	01 (02.0%)
	≥ 41	00 (00.0%)
**02.**	**Gender (** ***N*** ** = 171;100%)**	
	Male	82 (48.0%)
	Female	89 (52.0%)
**03.**	**Residential area of patients by province (** ***N*** ** = 171;100%)**	
	Western	59 (34.5%)
	Southern	22 (12.9%)
	Sabaragamuwa	17 (09.9%)
	North Western	15 (08.8%)
	Central	15 (08.8%)
	North Central	09 (05.3%)
	Uva	16 (09.4%)
	Eastern	10 (05.8%)
	Northern	08 (04.7%)

1 B.	Demographic and socioeconomic characteristics obtained from TS	
	Variable	Frequency (N = 90)
**4.**	**Ethnicity**	
	Sinhala	69 (76.7%)
	Tamil	10 (11.1%)
	Moor	11 (12.2%)
**5.**	**Parental age (years) at birth of index child**	
	**Father**	
	15–20	01 (01.1%)
	21–30	19 (21.1%)
	31–40	65 (72.2%)
	41–50	05 (05.6%)
	**Mother**	
	15–20	03 (03.3%)
	21–30	55 (61.1%)
	31–40	32 (35.6%)
	41–50	00 (00.0%)
**6**	**Parents’ educational status**	
**a.**	**Father**	
	Below General Certificate of Education (GCE) Ordinary level	14 (15.6%)
	Passed GCE Ordinary level	51 (56.7%)
	Passed GCE Advanced level	20 (22.2%)
	Tertiary education	05 (05.6%)
**b.**	**Mother**	
	Below GCE Ordinary level	10 (11.1%)
	Passed GCE Ordinary level	41 (45.6%)
	Passed GCE Advanced Level	26 (28.9%)
	Tertiary Education	13 (14.4%)
**7.**	**Parents’ Occupation** ^#^	
**a.**	**Father**	
	Unemployed	02 (02.2%)
	Skill level 1	07 (07.8%)
	Skill level 2	60 (66.7%)
	Skill levels 3 and 4	16 (17.8%)
	Armed forces	05 (05.6%)
**b.**	**Mother**	
	Unemployed/ Homemakers	72 (80.0%)
	Skill level 1	06 (06.7%)
	Skill level 2	06 (06.7%)
	Skill levels 3 and 4	06 (06.7%)
	Armed forces	00 (00.0%)
**8.**	**Total family income per month (Rupees)**	
	≤ 10,000	07 (07.8%)
	10,001–20,000	24 (26.7%)
	20,001–30,000	17 (18.9%)
	30,001–50,000	17 (18.9%)
	>Rs. 50,000	25 (27.8%)
**9.**	**Receiving financial assistance**	
	Yes	19 (21.1%)
	No	71 (78.9%)
**10.**	**Distance (km) from residence to LRH**	
	≤ 1	02 (02.2%)
	1.1–5	04 (04.4%)
	5.1–10	27 (30.0%)
	10.1–50	16 (17.8%)
	> 50	41 (45.6%)

#Occupations categorized according to international standard classification of occupation (ISCO-88) of the International Labour Organization: Skill level 1 - Elementary occupations; Skill level 2 - clerks, service workers and shop and market sales workers, skilled agricultural and fishery workers, craft and related trades workers, plant and machine operators and assemblers; Skill levels 3 & 4 - legislators, senior officials and managers, professionals, technicians and associate professionals

### Clinical features of the cohort of patients based on the information retrieved from the medical records at LRH and the telephone survey (Table 2)

#### Family history of RB (*N* = 171)

Among the total cohort of 171, a family history RB was for present in 5 (2.9%), including 1 (0.8%) unilateral and 4 (7.8%) bilateral patients.

#### Age of RB patients when first symptom/sign was observed (*N* = 90)

The highest proportion (26.7; *n* = 24) had the first symptom observed between 7 and 12 months of age. The second highest proportion (25.5%; *n* = 23) had their first symptom observed between 0 and 6 months.

#### Symptoms/signs at first visit to tertiary care unit (*N* = 171)

Ninety-eight (57.3%) patients presented with leukocoria, 28 (16.4%) with strabismus and 25 (14.6%) with both.

#### Duration between observing symptom/sign and first attendance at tertiary care unit (*N* = 90)

Median duration between observing the first symptom/sign and first attendance at the tertiary care unit was 8 weeks (IQR = 0–16) with a range of 0–24 weeks. There were 52 patients (57.8%) having a lag time of > 4 weeks (range 5–24 weeks).

#### RB stage (*N* = 171)

Among the total group of 171 patients, a majority (62.6%; *n* = 107) were in Group E and 25.7% (*n* = 44) in Group D. Among the unilateral patients, 69 (57.5%) were in Group E and 35 (29.2%) were in Group D. Among bilateral patients, 38 (74.5%) were in Group E for the first eye whereas none were in Group E for the second.

#### Parental consanguinity (*N* = 171)

The proportion of cases who reported parental consanguinity was 11.7% (*n* = 20).

#### Rates of enucleation (*N* = 171)

Of the total sample of 171, the number of enucleations was 121 (70.8%). Among unilateral and bilateral cases, the proportions were 69.2% (*n* = 83) and 74.5% (*n* = 38) respectively.

#### Modalities of RB therapy (*N* = 171)

Among the 121 (70.8%) patients who underwent enucleation, 46 (38% = 46/121) had undergone primary enucleation whereas 75 (43.9%) patients had undergone chemo, laser or cryotherapy prior to secondary enucleation. Salvage of the eye was possible for 50 (29.2%) patients following non-surgical treatment, including systemic and intravitreal chemotherapy and laser therapy.

#### Mortality rate (*N* = 171)

The total number of deaths was 2 (1.2%).

**Table 2:** Clinical features obtained from the medical records (MR) from the Lady Ridgeway Hospital (LRH) and the telephone survey (TS)

No.	Clinical features obtained from MR at LRH		
	Variable		Frequency
**1.**	**Family history of RB**		
	**Total sample (** ***N*** ** = 171; 100%)**		
	Yes		005 (02.9%)
	No		166 (97.1%)
	**Unilateral (** ***N*** ** = 120; 70.2%)**		
	Yes		001 (00.8%)
	No		119 (99.2%)
	**Bilateral (** ***N*** ** = 51; 29.8%)**		
	Yes		04 (07.8%)
	No		47 (92.2%)
**2.**	**Age (months) of RB patients when symptom/sign was first observed (** ***N*** ** = 90; 100%)**		
	00–06		23 (25.5%)
	07–12		24 (26.7%)
	13–18		15 (16.7%)
	19–24		07 (07.8%)
	25–30		07 (07.8%)
	31–36		02 (02.2%)
	≥ 37		12 (13.3%)
**3.**	**Symptoms/signs at first presentation (** ***N*** ** = 171; 100%)**		
	Leukocoria		98 (57.3%)
	Strabismus		28 (16.4%)
	Leukocoria and strabismus		25 (14.6%)
	Others*		08 (04.8%)
	Missing data		12 (07.0%)
**4.**	**Duration (weeks) between first observing symptom/sign & first attendance at tertiary care unit (** ***N*** ** = 90; 100%)**		
	≤ 01		31 (34.4%)
	> 01–04		07 (07.8%)
	05–08		20 (22.2%)
	09–12		04 (04.4%)
	≥ 13		28 (31.1%)
**5.**	**Clinical staging of disease**		
**a**.	**Total group (** ***N*** ** = 171; 100%)**		
	Group A		002 (01.2%)
	Group B		015 (08.8%)
	Group C		003 (01.7%)
	Group D		044 (25.7%)
	Group E		107 (62.6%)
**b.**	**Unilateral (** ***N*** ** = 120; 70.2%)**		
	Group A		02 (01.7%)
	Group B		11 (09.2%)
	Group C		03 (02.5%)
	Group D		35 (29.2%)
	Group E		69 (57.5%)
**c.**	**Bilateral (** ***N*** ** = 51; 29.8%)**		
**i.**	**First eye**		
	Group A		00 (00.0%)
	Group B		04 (07.8%)
	Group C		00 (00.0%)
	Group D		09 (17.6%)
	Group E		38 (74.5%)
**ii.**	**Second eye**		
	Group A		12 (23.5%)
	Group B		11 (21.6%)
	Group C		01 (02.0%)
	Group D		27 (52.9%)
	Group E		00 (00.0%)
**6.**	**Parental consanguinity (** ***N*** ** = 171)**		
	Yes		020 (11.7%)
	No		151 (88.3%)
**7.**	**Undergone enucleation**		
**a.**	**Total sample (171; 100%)**		
	Yes		121 (70.8%)
	No		050 (29.2%)
**b.**	**Unilateral (** ***N*** ** = 120)**		
	Yes		83 (69.2%)
	No		37 (30.8%)
**c.**	**Bilateral (** ***N*** ** = 51)**		
	Yes		38 (74.5%)
	No		13 (25.5%)
**8.**	**Modalities of RB therapy (** ***N*** ** = 171; 100%)**		
	Primary enucleation		46 (26.9%)
	Secondary enucleation		75 (43.9%)
	Ocular salvage therapy		50 (29.2%)
**9.**	**Mortality (** ***N*** ** = 171; 100%)**		
	Yes		002 (01.2%)
	No		168 (98.2%)

*Others (Symptoms at first referral) include visual inattention, upper lid lump, red eye, hazy cornea, total retinal detachment, glaucoma, uveitis, proptosis, large eye ball, white reflex and hyphema)

### Correlates related to lag time between first observation of symptom/ sign and first attendance at the tertiary care unit (Table 3)

#### Bivariate analysis

Fifty eight percent (57.8%; *n* = 52) had a lag time of > 4 weeks in comparison to 42.2% (*n* = 38) with a lag time ≤ 4 weeks (Table 2). However, none of the variables analyzed reached statistical significance. The odds ratios of gender and ethnicity were close to 1.0.

The odds ratios for lower parental educational status, parents being engaged in low skilled occupations, not receiving financial assistance and a distance ≥ 10 km from residence to LRH were all < 1.0. These factors therefore had a tendency to be protective against a lag period of > 4 weeks in obtaining treatment at the tertiary care unit (Table 3).

#### Multivariable analysis

None of the variables in the model reached statistical significance. However, the variables in the final equation included were mothers’ low skilled occupations (OR 0.28 (95% CI [0.031, 2.55]; *p* = .260) and fathers’ educational status < GCE O/L (OR 0.530 (95% CI [0.17, 1,69]; *p* = .284) both which have the tendency to be protective against a lag period of > 4 weeks.

**Table 3:** Bivariate analysis to determine correlates for lag time of > 4 weeks between first observation of symptom/sign and first attendance at tertiary care unit

No.	Variable	Lag Time		Chi-square test	Odds Ratio
		(*N* = 90)		df = 1	(95% CI)
		> 4 weeks	≤ 4 weeks	Probability	
		***n*** ** = 52 (58%)**	***n*** ** = 38 (42%)**		
**1.**	**Age of RB patients of TS at first visit to LRH**				
	> 20 months	21 (40.4%)	11 (28.9%)	0.266	1.66
	≤ 20 months	31 (59.6%)	27 (71.1%)		(0.68–4.06)
**2.**	**Gender**				
	Male	30 (57.7%)	22 (57.9%)	0.985	0.99
	Female	22 (42.3%)	16 (42.1%)		(0.42–2.33)
**3.**	**Ethnicity**				
	Sinhala	40 (76.9%)	29 (76.3%)	0.947	1.03
	Tamils + Moors	12 (23.1%)	09 (23.7%)		(0.37–2.81)
**4.**	**Mothers’ educational status** ^**#**^				
	< GCE O/L	04 (07.7%)	06 (15.8%)	0.230	0.44
	≥ GCE O/L	48 (92.3%)	32 (84.2%)		(0.10–1.77)
**5.**	**Fathers’ educational status** ^**#**^				
	< GCE O/	06 (11.5%)	08 (21.1%)	0.221	0.49
	≥ GCE O/L	46 (88.5%)	30 (78.9%)		(0.15–1.60)
**6.**	**Mothers’ occupation** ^**##**^				
	Unemployed + SL* 1&2	47 (90.4%)	37 (97.4%)	0.192	0.25
	SL 3&4 + Armed forces	05 (09.6%)	01 (02.6%)		(0.01–1.96)
**7.**	**Fathers’ occupation** ^**##**^				
	Unemployed + SL* 1&2	39 (75.0%)	30 (78.9%)	0.664	0.80
	SL 3&4 + Armed forces	13 (25.0%)	08 (21.1%)		(0.28–2.191)
**8.**	**Total family income (Rupees per month)**				
	≤ 30,000	30 (56.6%)	18 (47.4%)	0.335	1.52
	> 30,000	22 (43.4%)	20 (52.6%)		(0.65–3.55)
**9.**	**Financial assistance received**				
	No	40 (76.9%)	31 (81.6%)	0.595	0.75
	Yes	12 (23.1%)	07 (18.4%)		(0.25–2.15)
**10.**	**Distance (km) from residence to LRH (km)**				
	> 10.0	48 (92.3%)	36 (94.7%)	0.650	0.67
	≤ 10.0	04 (07.7%)	02 (05.3%)		(0.08–3.98)

#GCE O/L- General Certificate of Education Ordinary Level

##Occupations categorized according to international standard classification of occupation (ISCO-88) of the International Labour Organization: Skill level 1 - Elementary occupations; Skill level 2 - clerks, service workers and shop and market sales workers, skilled agricultural and fishery workers, craft and related trades workers, plant and machine operators and assemblers; Skill levels 3 & 4 - legislators, senior officials and managers, professionals, technicians and associate professionals and armed forces

*SK- Skill level

## Discussion

Data from this Sri Lankan study shows that 88.3% of the 171 RB cases had groups D and E tumours at presentation. Despite this high rate of locally advanced disease, the observed death rate was low, at 1.2%. However, there was a high (71%) rate of enucleations. The data also indicated that more than half (58%) of RB cases had a lag time of > 4 weeks between first observation of a symptom/sign to first attendance at the tertiary care unit.

The low mortality rate could be attributed to the availability of a designated tertiary care unit which provides standardized healthcare with practice of evidence-based medicine. The fact that all the variables included in the bivariate and multivariable analyses did not reach statistical significance (with high probability levels) could be due to inadequate sample size. However, the 95% confidence intervals for some of the variables were relatively narrow, indicating reasonable precision, which would negate the argument of inadequate sample size.

Of the ten variables subjected to bivariate analysis (all of which were not significant), two variables (age of RB patients at first visit to LRH and monthly family income) had odds ratios of more than 1.0, which indicates a tendency towards being risk factors for having a delayed lag time of > 4 weeks. Gender and ethnicity had odds ratios which were almost one and the remainder demonstrated a tendency towards being protective factors in favour of a lag time of < 4 weeks. According to Visintainer (2021), there are some useful observations that one can make from studying the effect measure and its 95% confidence interval, despite statistical significance [13]. Latter was used to decide the adequacy of the sample size based on its width. The effect measure indicates the strength and direction of the association between exposure and outcome variables, based on which probable risk/protective status was determined.

Sri Lanka is currently classified as a lower-middle income country (LMIC) by the World Bank [14]. According to Chintagumpala (2019) [15], the incidence of RB is higher in LMICs. Fabian et al. (2018) [16] had stated that there are no gender, ethnic, environmental or socioeconomic factors that are associated with RB, as the incidence of it is uniform across the whole world. On the contrary, they claim that over 80% of the RB cases are from the LMICs. However, according to Jain et al. (2019) [17], based on population estimates of six countries in the Asia–Pacific region, the burden of RB had been reported as 43%, which is much lower than the incidence reported above.

The percentage of cases who present with locally or systemically advanced stages of disease is also said to be higher in LMICs. This is attributed to prolonged time to diagnosis (TTD)/lag time from initial detection of symptom/sign. Factors associated with TTD/lag time include low socioeconomic status, lack of immediate access to medical care and lower level of parental education, all of which are common in LMICs [15]. With regard to the present study, lower parental educational status and socioeconomic indicators (except monthly income) emerged as protective factors (tendency to reach tertiary care services within four weeks of detecting the first symptom/sign) even though these were not statistically significant. Sri Lanka has accessible and quality healthcare for all, which is free at the point of delivery and accessible even to those in more remote parts of the island [18]. Therefore, unlike in other LMICs, lack of access to the tertiary care in Sri Lanka could not be considered as a reason for greater lag time.

According to Posner et al. (2017) [19] the TTD/lag time occurs due to three delays: lag 1 is due to parental delay in consulting a primary care medical officer, lag 2 is due to delay of the primary care medical officer making a referral to the ophthalmologist (health professional delay) and lag 3 is the delay due to time of ophthalmologist to refer to the RB tertiary care unit/center. The exact reason for occurrence of longer lag time in Sri Lanka is uncertain, and this warrants further investigations. In the UK, the recommended time to consult the specialist after detecting the first symptom is within two weeks [20] Although delays in diagnosis were suspected of being linked to high-risk RB in the UK, no correlation between the two have been observed [19, 20].

As stated by Chintagumpala (2019) [15], available information on RB is mostly based on retrospective studies. The respondents included in the telephone survey in this study were required to recall past events which occurred up to eight years previously. Despite some events in life being recalled accurately, it may not be true for recall of the lag time between observation of the initial symptom to seeking tertiary care services. This may be compounded by providing socially desirable responses, especially when it is not possible to recollect past events precisely. Thus, the reliability of the information provided may be considered debatable. Lack of this information, which is required for planning of interventions related to increasing awareness among primary health care providers and the general public is a major limitation of this study. This is overcome by conducting prospective surveys where recall issues of parents can be minimized.

In this cohort, the first observed sign by parents was leukocoria (72%) similar to that reported by Pandey (2014) [21] and Chithungumpala et al. (2007) [4] whereas the second most common symptom was strabismus [4], similar to that in the present study. Both these symptoms appear at more advanced stages of disease, where salvage of eyes and preservation of vision is difficult. Diagnosis of RB in children presenting with leukocoria and strabismus correlate well with patient survival but not with ocular survival rates [22]. Therefore, ocular salvage and vision preservation ideally requires recognition of disease before occurrence of leukocoria [22]. The red pupillary reflex (RPR) is gaining recognition as a primary care level screening method [23]. In Sri Lanka, newborn examination is conducted by doctors in the postnatal ward. The mandatory examining for RPR needs to be strictly implemented during examination of the newborns as well as on a routine basis at the community level by the Medical Officers of Health [MOH]. Lack of awareness has been reported as one of the main reasons for not consulting a doctor early [7] and the positive impact of educating the community and training of health professionals has been reported by Al-Nawaiseh et al. (2017). [24]. It is therefore essential to increase parents’ awareness about the need to have RPR testing and seeking medical advice soon after detecting the first RB sign.

Gender proportions reported were 48% for males and 52% for females [1:1.1] for the cohort of 171 in the present study. Lack of gender predisposition had been reported by Andreoli et al. (2017) [25] and Gupta et al. (2020) [26] in reference to a North Indian study conducted from 2009 to 2018. In addition, survival rates also had shown no gender difference according to Andreoli et al. (2017) [25]. However, a review conducted by Singh et al. (2017) in North India from 1998 to 2014 reported that 62% of the RB cases were males. This may be attributed to socioeconomic and cultural factors disadvantaging female children in settings with inadequate resources [27]. Lack of gender predilection is confirmed by the results of the present study too, where the odds ratio for the association between gender and lag time for attending the tertiary care unit was close to one.

Chintagumpala et al. (2007) [4] have reported that 20–30% of patients have bilateral RB, which is consistent with the results of the present study (29.8%) [4]. Among the cohort of 171 patients, only 3% (*n* = 5) reported a positive family history and among those five patients, one had a unilateral and the remainder, bilateral RB. Pandey (2014) [21] has reported 6% to be familial, which may be considered as consistent with the present study. In those with a positive family history, the risk of siblings being affected is high, emphasizing the need for genetic counselling and testing in addition to careful screening of affected children and their siblings [21, 28].

Results from this study have indicated that a majority of RB patients presented with locally advanced groups D and E tumours, with 71% undergoing enucleations. Saving life, rather than preservation of the eye, is the main goal of treatment in advanced stages of this disease [16, 21] and this is consistent with current, international RB management protocols. A similar proportion (72%) of enucleations have been reported from the study conducted by Bourkiza et al. (2020) [6] in patients treated between January 2006 to December 2011 in the UK, and a higher proportion of 87.5% in the study conducted in a tertiary care unit in North India between 2009 and 2018 [16]. Lag time to RB diagnosis from the identification of the initial symptom/sign is considered as a salient determinant of advanced disease [15].

The globe salvage rate in Sri Lanka (29%) is similar to other LMICs (34%) whereas the globe salvage of high-income countries is around 70%. There is a reported positive correlation between globe salvage and lower Gini index [29]. The latter measures the income distribution of a population on a scale which ranges from 0 (0%) – 1(100%). A value of zero indicates perfect equality with lower measurements being observed in high-income countries.

In the LMICs countries such as in most parts of Asia, Africa and South America, the mortality rates range from 20 to 70% [2, 16, 26]. In comparison, in this Sri Lankan study, it was 1.2%. This low mortality rate is comparable to that of high-income countries of Europe and North America, where mortality rates range from 3 to 5% [30]. The two deaths mentioned above were due to patients undergoing alternative, traditional therapy after initial RB diagnosis, and presenting later with metastatic disease. The low mortality reported here may be attributed to the delivery of care at a single, designated tertiary care centre for RB in Sri Lanka as stated previously.

This study has identified a disproportion related to distribution of cases by provinces in Sri Lanka, with two provinces (Western and Uva) having rates which were double that of the lowest rate reported. The population in the Western province is six times that of Uva, whereas Western and Uva are comparable with regard to gender distribution. Comparing the Gini coefficient (household and per person), both provinces are almost similar. However, with regard to educational attainment and mean income per household per month, Uva is well below the Western province [31]. The studies from different parts of the world, as well as from within the same region have shown wide variations with regard to determinants of RB, its clinical presentation and survival outcomes of children affected by the disease [17]. In addition, Posner et al. (2017) [19] and Chinthagumpala et al. (2007) [4] have stated that the cause of the higher case rates in the LMICs cannot be explained.

Despite the availability of the telephone numbers of all 171 RB patients in the medical records, the telephone survey was only able to contact 53% of those patients. The low response rate is a limitation of this study, as information pertinent to lag time in seeking tertiary care services among non-respondents would have been excluded, potentially leading to a selection bias. A prospective survey would minimize such bias.

The available medical records in the LRH and also copies in the patients’ notes are mainly paper based and hand written. Data collection and recording may become inconsistent in this situation. The use of customized electronic or paper based medical record templates may reduce the problems of data gathering in the future.

The current recommendation for RB management is for centralization of tertiary care services, with development of multidisciplinary teams of experts to improve treatment outcomes. The latter requirement has already been fulfilled in Sri Lanka, and the services are provided free of charge throughout their stay in the RB unit. However, the data from RB patients in Sri Lanka indicates that accessing the services imposes financial burdens for the affected families, especially those travelling long distances to attend clinics in Colombo. This includes loss of daily income for one or both parents, costs of transport and for boarding or lodging in Colombo. In this study, 53% of the families were earning < Rs. 30,000 and only one fifth were receiving donations. As this variable was considered a probable risk factor with an odds ratio of 1.5 (a factor contributing to lag time greater than four weeks), even though not statistically significant, it is unethical to disregard this finding. Therefore, it is imperative that at least those low-income families, who live long distances away from Colombo, be provided with some financial assistance. Availability of a state subsidized programs or community-based private agencies that can support these families with their financial needs. This would help to alleviate the financial burden that is currently borne by the families or by borrowing money from relatives/friends/employers and may be finally end up being permanently indebted to them.

## Conclusion

The age at diagnosis of both unilateral and bilateral RB cases and the proportion of unilateral cases in this study were similar to data from other reported series. Seventy one percent of patients had to undergo enucleation on account of advanced stage of disease at presentation. Having a centralized tertiary care unit is probably pivotal in achieving a low mortality rate. Lag time in seeking medical care extended up to six months based on the telephone survey, which needs to be addressed by conducting culturally relevant and economically feasible awareness programs for parents/general public. In addition, providing medical information and psychological support for the affected parents should be organized. It is also crucial to conduct universal screening for early identification of RB cases on a regular basis according to local and international recommendations. Implementation of effective screening strategies on a routine basis through trained primary care physicians and field public health staff, through the existing domiciliary care services is a fundamental requisite. As future research, it is necessary to plan and conduct prospective studies using larger samples in order to bridge the existing knowledge gaps related to RB.

### Electronic supplementary material

Below is the link to the electronic supplementary material.

Supplementary Material 1

Supplementary Material 2

## Data Availability

The datasets used and/or analysed during the current study are available from the corresponding author on reasonable request.

## Abbreviations

CI: Confidence interval
Df: Degrees of freedom
GCE O/L: General certificate of education ordinary level
GDP: Gross domestic product
IIRC: International Intraocular Retinoblastoma Classification
IQR: Interquartile range
ISCO: International standard classification of occupation
LMIC: Low- and middle-income countries
LRH: Lady Ridgeway hospital
MOH: Medical Offices of Health
OR: Odds ratio
p: Probability
RB1: Retinoblastoma gene
RB: Retinoblastoma
