# Supplementary material for: Retinoblastoma patients treated in Sri Lanka from 2014 to 2020: epidemiology, clinical status and correlates of lag time in seeking tertiary care services
Source: BMC Ophthalmol. 2024 Jul 17;24:292. doi: 10.1186/s12886-024-03541-3 (PMC11256412; doi:10.1186/s12886-024-03541-3)
Supplement: Supplementary file 1 — Supplementary Material 1 [file 12886_2024_3541_MOESM1_ESM.docx]

**Additional file 1**

Data No:

Date:

**Retinoblastoma Data Collection Sheet**

**General Information**

| Name: | Sex: M/ F: |
| --- | --- |
| DOB: | Age at review:  Ht OFC Wt.  Centile |

**Birth History Antenatal Problems**

| Term delivery : | Non recorded : |
| --- | --- |
| 36-40 weeks: | If recorded, Specifiy: |
| 32-35 weeks: |  |
| < 31 weeks: | Apgars:  1 min 5 min 10min |

| **Specify Gestation:** |
| --- |

**Mode of delivery Birth**

| SVD: | Weight: |
| --- | --- |
| Forceps: | Length: |
| LSCS: | OFC: |

**Neonatal problems**

|  |
| --- |

**Clinical featuers**

| Age / date of presentation: |
| --- |
| **Clinical featues identified at presentation**  Leukocoria : Yes / No  Strabimus : Yes / No  Red painful eye with glaucoma: Yes / No  Poor vision : Yes / No  Orbital cellulitis : Yes / No  Unilateral mydriasis : Yes / No  Heterochromia iridis : Yes / No  Hyphema : Yes / No  Other |
| Symptoms identification (How long): Identified by Parents:  Identified by Doctors: |
| Diagnosis (International classification)  Group Stage  Right  Left |

**Retinoblastoma clinical findings:**

Unilateral / Multifocal/ Bilateral

First tumour: diagnosis date

Second tumour: diagnosis date

Others including pineal involvement:

**Treatment**

| Radiation Therapy : Yes / No |
| --- |
| Enucleation of eye : Yes / No |
| External beam radiotherapy : Yes / No |
| Laser photocoagulation : Yes / No |
| Cryotherapy : Yes / No |
| Systemic chemotherapy : Yes / No How many Cycles. 1. JOE chemo:  2. High risk chemo: |
| Intravitreal chemotherapy : Yes / No |

| **Surgery:**  **Date of Surgery:** |
| --- |

**Pathology status:**

| Pathology Number: |
| --- |
| Date: |
| Histology report:  Retinoblastoma confirmed:  Adverse histology features   1. Involvement of optic nerve 2. Involvement of choroid (Uveal tract) 3. Involvement of anterior chamber (Vitreous seeding) |
|  |
| Follow up plan after surgery:  After Radiation: |

**Family History:**

| Parents name  Mother:  Father: | DOB:  Mother:  Father: |
| --- | --- |
| Parents eye examination:  Mother: Yes / No Findings  Father: Yes / No Findings | Non Recorded: |
|  | If Recorded Specify; |
| Siblings tested  Name:  Age of last examination  Findings Normal/ Rb features  Details:  Name:  Age of last examination  Findings Normal/ Rb features  Details:  Name:  Age of last examination  Findings Normal/ Rb features  Details:  Name:  Age of last examination  Findings Normal/ Rb features  Details: | Non Recorded: |
|  | If Recorded Specify; |

**Pedigree: Add any other relevant family history details**
