# Supplementary material for: Retinoblastoma patients treated in Sri Lanka from 2014 to 2020: epidemiology, clinical status and correlates of lag time in seeking tertiary care services
Source: BMC Ophthalmol. 2024 Jul 17;24:292. doi: 10.1186/s12886-024-03541-3 (PMC11256412; doi:10.1186/s12886-024-03541-3)
Supplement: Supplementary file 2 — Supplementary Material 2 [file 12886_2024_3541_MOESM2_ESM.docx]

**Additional file 2**

Data No:

Date:

**Retinoblastoma Data Collection Sheet 2**

**General Information**

| Name: | Sex: M/ F: |
| --- | --- |
| DOB: | Current address (town/ district) |

**Family History:**

| Parents name and DOB:  Mother:  Father: |
| --- |
| Age at diagnosis by parents:  Age at diagnosis by Doctor: |
| Ethnicity: Sinhalese  Tamil  Muslim  Other |
| Education: Father  Mother |
| Profession: Father :  Employer:  Mother:  Employer: |
| Father’s job Income: <10,000  10,000 – 20,000  20,000 – 30,000  30,000 – 50,000  >50,000  Mother’s job Income: <10,000  10,000 – 20,000  20,000 – 30,000  30,000 – 50,000  >50,000  Additional source of income : Y/N Amount: <10,000  10,000 – 20,000  20,000 – 30,000  30,000 – 50,000  >50,000 |
| Help from government: eg Sanurdhi  Help from charities:  Help from family: |
| Distance from home to nearest Hospital:  Distance from home to LRH  Do they own a vehicle? Y/N  If yes- what? Specify (car/ three wheeler/ motor cycle/ bicycle/ lorry)  How they come to LRH: |
| If using public transport  How do they travel to the nearest bus/ train station:  Distance from home to the nearest bus/ train station:  How long does it take to come to LRH? |
| Cost per visit for transport:  Other costs of coming to hospital  Food  Lodgings  other  How many visits per month/ year since the diagnosis of Rb? |
| Time/ Day spent per visit:  1^st^ visit  How many visits after this: |
| Number of Siblings:  Younger: Male: Female:  Elder: Male: Female: |
| Who is accompanying the child to the hospital:  Who is looking after the other children:  Age of the person who is looking after the other child: |
| Parents eye examination:  Mother: Yes / No Findings  Father: Yes / No Findings |
|  |
| Siblings tested  Name:  Age of last examination  Findings Normal/ Rb features  Details:  Name:  Age of last examination  Findings Normal/ Rb features  Details:  Name:  Age of last examination  Findings Normal/ Rb features  Details: |
|  |
